# The EcoCyc Database in 2021

**DOI:** 10.3389/fmicb.2021.711077

**Published:** 2021-07-28

**Authors:** Ingrid M. Keseler, Socorro Gama-Castro, Amanda Mackie, Richard Billington, César Bonavides-Martínez, Ron Caspi, Anamika Kothari, Markus Krummenacker, Peter E. Midford, Luis Muñiz-Rascado, Wai Kit Ong, Suzanne Paley, Alberto Santos-Zavaleta, Pallavi Subhraveti, Víctor H. Tierrafría, Alan J. Wolfe, Julio Collado-Vides, Ian T. Paulsen, Peter D. Karp

**Affiliations:** ^1^Bioinformatics Research Group, Artificial Intelligence Center, SRI International, Menlo Park, CA, United States; ^2^Centro de Ciencias Genómicas, Universidad Nacional Autónoma de México, Cuernavaca, México; ^3^Department of Molecular Sciences, Macquarie University, Sydney, NSW, Australia; ^4^Instituto de Energías Renovables, Universidad Nacional Autónoma de México, Temixco, México; ^5^Department of Microbiology and Immunology, Stritch School of Medicine, Loyola University Chicago, Maywood, IL, United States; ^6^Department of Biomedical Engineering, Boston University, Boston, MA, United States

**Keywords:** *Escherichia coli*, EcoCyc, model-organism database, drug efflux transporters, metabolism, gene regulation

## Abstract

The EcoCyc model-organism database collects and summarizes experimental data for *Escherichia coli* K-12. EcoCyc is regularly updated by the manual curation of individual database entries, such as genes, proteins, and metabolic pathways, and by the programmatic addition of results from select high-throughput analyses. Updates to the Pathway Tools software that supports EcoCyc and to the web interface that enables user access have continuously improved its usability and expanded its functionality. This article highlights recent improvements to the curated data in the areas of metabolism, transport, DNA repair, and regulation of gene expression. New and revised data analysis and visualization tools include an interactive metabolic network explorer, a circular genome viewer, and various improvements to the speed and usability of existing tools.

## Introduction

*Escherichia coli* is the most well-studied bacterial model organism. The scientific literature reports on more than a century of research on *E. coli*, including paradigm-shifting research on enzyme function, gene regulation and genetic engineering. Knowledge gained about the biology of *E. coli* is often the basis for assigning gene product functions in less studied organisms, and scientists turn to the body of *E. coli* research to begin to understand these functions in the context of their organism of interest. However, despite the long history of research, the functions of a surprising number of *E. coli* gene products remain unknown (Ghatak et al., 2019). Knowledge gaps remain even in areas that have been studied for decades, and the genes of unknown function that are essential for growth in rich media exist.

The EcoCyc database has been manually curated by PhD-level scientists for nearly three decades (Karp and Riley, 1993; Keseler et al., 2017), and its coverage has been expanded from metabolism to the entire genome. Extensive literature searches enable curators to capture both established knowledge and new insights. Perhaps equally important, the curation process can capture a lack of knowledge *via* the assignment of detailed evidence codes. For example, the participation of an enzyme in a metabolic pathway is often established by assaying its biochemical function *in vitro*, resulting in an IDA (inferred from direct assay) evidence code. Occasionally, an enzyme’s function within a metabolic pathway is known only by its mutant phenotype, resulting in an IMP (inferred from mutant phenotype) evidence code. Therefore, EcoCyc provides an overview of current knowledge and serves as a resource for the identification of knowledge gaps.

EcoCyc collects research conducted with the laboratory workhorse K-12 strains projected on the genome sequence of the first sequenced *E. coli* K-12 strain, MG1655. Many other *E. coli* strains have been sequenced since that first genome sequence. To leverage the EcoCyc curation effort and enhance the quality and usability of all *E. coli* databases within the BioCyc database collection (of which EcoCyc is a member database), curated gene and protein data have also been propagated from EcoCyc to orthologs in databases for 480 other *E. coli* strains *via* a new automated method (Paley et al., 2021). In this article, we highlight and summarize additions to the data content and improvements to search, data-analysis, and visualization tools since our last publication reporting on updates to EcoCyc (Keseler et al., 2017).

## Results

### Curated Data in EcoCyc

An overview of many of the data types captured in EcoCyc version 24.5, released on January 7, 2021, is shown in Table 1. This section highlights some notable updates since release version 21.1 (Keseler et al., 2017).

**Table 1:** Select EcoCyc content and *Escherichia coli* gene product functions in release 24.5.

Data type	Number of database objects
Genes	4,518
Gene products covered by a mini-review	4,087
Gene products with GO terms with EXP evidence	3,494
Enzymes	1,682
Metabolic reactions	2,151
Compounds	3,023
Transporters	288
Transport reactions	527
Transported substrates	375
Transcription factors	213
Transcription factor binding sites	4,076
Regulatory interactions	8,631
Transcription initiation	5,505
Transcription attenuation	24
Regulation of translation	318
Enzyme modulation	2,763
DNA Sites
Transposons	50
REP elements	697
Cryptic prophages	12
Literature citations	39,865

#### Metabolism

EcoCyc integrates historical data with the most recent insights from the published literature. For example, the enzymes involved in the biosynthesis of ubiquinol-8 were genetically identified decades ago. The current representation of this pathway in EcoCyc can be seen by following this link: https://ecocyc.org/ECOLI/NEW-IMAGE?type=PATHWAY&object=PWY-6708&detail-level=2.

For most of the enzymes, curators were unable to find the published reports of biochemical assays of the activities of ubiquinol-8 biosynthesis enzymes, which is likely due to the general difficulty of, lack of interest in, and/or obstacles to publishing negative data. The unavailability of this information highlights the importance of recording the lack of specific types of data, as is being done in EcoCyc: the evidence codes associated with many of the individual enzymatic reactions in this pathway remain at the “inferred by mutant phenotype” level.

This lack of biochemical data seemed surprising, because most sof the enzymes in ubiquinol-8 biosynthesis, like those in menaquinol-8 biosynthesis,^1^ are located in the cytoplasm. However, unlike menaquinol-8 biosynthesis, where the hydrophobic octaprenyl tail is added late in the pathway by the inner membrane-localized enzyme MenA, mutant phenotype data showed that the octaprenyl tail of ubiquinol-8 is added early in the pathway. Also, two accessory factors with no predicted biochemical function, UbiJ and UbiK, were identified only by their mutant phenotypes (Aussel et al., 2014; Agrawal et al., 2017; Loiseau et al., 2017). The puzzle pieces fell into place in 2019, when Hajj Chehade et al. discovered that most of the ubiquinol-8 biosynthetic enzymes and the two accessory factors form a soluble complex (metabolon) in the cytoplasm. This complex is able to perform the biochemical transformations while shielding the octaprenyl tail from the aqueous environment (Hajj Chehade et al., 2019). However, other questions remain. The UbiB protein is involved in ubiquinol-8 biosynthesis based on a mutant phenotype. It was originally thought to provide a catalytic activity within the pathway (Cox et al., 1969), but is now proposed to function as a regulator (Poon et al., 2000; Hajj Chehade et al., 2013). Each of these pieces of data can be accessed in multiple ways, for example, by hovering over enzyme names to show the evidence codes associated with their functions and by reading the free-text summaries for the pathway and each enzyme.

#### Transmembrane Transport

**Figure 1:**
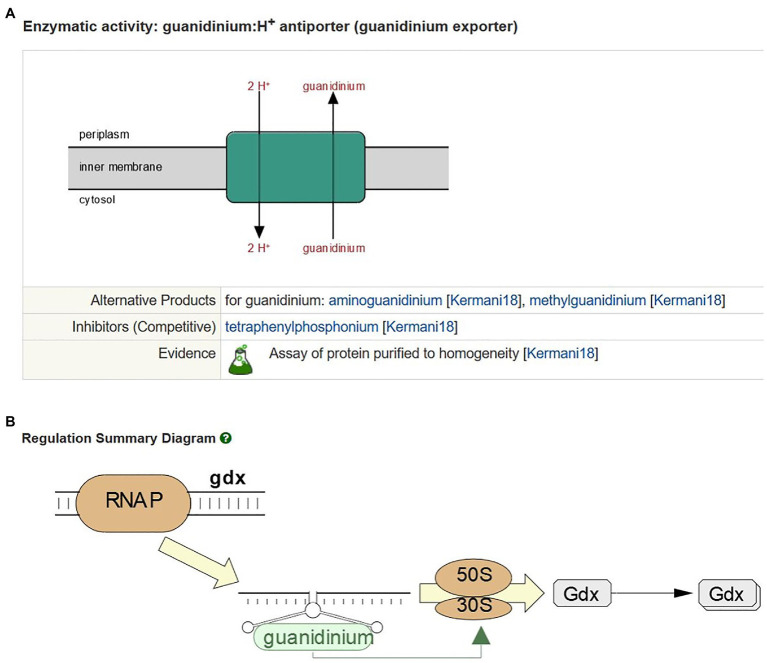
Curation of Gdx in EcoCyc. **(A)** The guanidinium:proton antiport reaction mediated by Gdx. **(B)** Gdx regulation by a guanidine II riboswitch.

Newly characterized transporters reported in the literature remain a focus for curation. Recent highlights include the curation of the pyruvate:proton symporters BtsT (Kristoficova et al., 2018) and CstA (Hwang et al., 2018; Gasperotti et al., 2020), the Zn^2+^:proton symporter ZntA (Gati et al., 2017), and a guanidinium:proton antiporter Gdx (Kermani et al., 2018). The latter transporter is regulated by a guanidine-II riboswitch predicted to act as a translation “on” switch (Huang et al., 2017; Sherlock et al., 2017). As part of the curation process, the gene names and free-text summaries for these proteins were updated, and transport reactions (Figure 1A) and regulatory information (Figure 1B) were added.

The guanidinium:proton antiporter Gdx is a member of the small multidrug resistance (SMR) family of proton-dependent drug efflux transporters. EcoCyc currently represents 25 known energy-dependent drug efflux transporters, including representatives from five of the seven major families of efflux transporters (Chitsaz and Brown, 2017). We have reviewed and updated the curation of all the drug efflux transporters in EcoCyc and improved our representation of the specific substrates, both physiological and non-physiological, that are exported by these proteins. Many new reactions and compounds have been added to the database as a result of this update. Readers interested in this area can view a freely available SmartTable of all drug efflux transporters and their reactions at the following link: https://ecocyc.org/group?id=biocyc14-4655-3823813233.

#### DNA Repair

**Figure 2:**
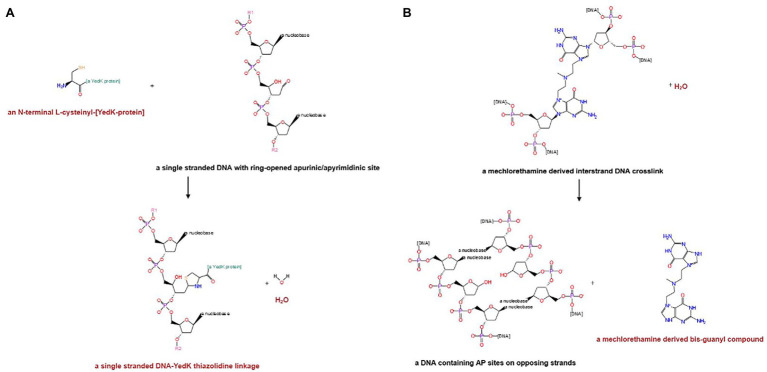
Reactions assigned to recently characterized DNA repair proteins in EcoCyc. **(A)** Genome maintenance protein YedK. **(B)** Interstrand DNA crosslink repair glycosylase YcaQ.

Significant improvements have been made to the curation of DNA repair enzymes, with a particular focus on the addition of reactions that accurately reflect the catalytic activities of these important proteins. Eleven new reactions were created as part of this process, including those for two newly described enzymes: the genome maintenance protein encoded by *yedK* (Mohni et al., 2019; Thompson et al., 2019; Wang et al., 2019) and an interstrand DNA crosslink repair glycosylase encoded by *ycaQ* (Bradley et al., 2020). Figure 2 shows the new reactions assigned to YedK and YcaQ.

#### Lysine Acetylation Sites

Protein N^ε^-lysine acetylation is a common post-translational modification, resulting from transfer of an acetyl group (CH_3_CO) to the ε-amino group (N-ε) of lysine residues within a protein. Acetylation increases the side-chain size and neutralizes the positive charge of the lysine residue, potentially altering protein activity (Christensen et al., 2019). Some proteins regulated by N^ε^-lysine acetylation include the central metabolic enzymes acetyl-CoA synthetase (Starai and Escalante-Semerena, 2004), enolase (Nakayasu et al., 2017), and malate dehydrogenase (Venkat et al., 2017), as well as the transcription factors PhoP (Ren et al., 2019) and CRP (Davis et al., 2018). N^ε^-lysine acetylation can be catalyzed by lysine acetyltransferases (KATs) using acetyl-CoA as the acetyl donor. The best studied KAT in *E. coli* is YfiQ (also known as Pat, PatZ, and Pka). Recently, four novel KATs – YjaB, YiaC, RimI, and PhnO – were revealed (Christensen et al., 2018). N^ε^-lysine acetylation can also occur without the help of a dedicated enzyme; in this case, the acetyl donor is acetyl phosphate, a high energy central metabolic intermediate that accumulates when carbon is in excess (Weinert et al., 2013; Kuhn et al., 2014; Christensen et al., 2017).

We greatly expanded the coverage of lysine acetylation in EcoCyc by importing five acetylome datasets that identify specific lysine positions in proteins that have been subject to acetylation (Kuhn et al., 2014; Schilling et al., 2015; Christensen et al., 2018). The lysine acetylation sites are recorded and displayed as protein features. When visiting a protein page, clicking on the tab “Protein Features” will show the amino acid sequence and a table of annotations that indicate specific sites or regions with evidence for a variety of functional properties including known acetylation sites. Two examples can be found by following these links for proteins AceF and LipA, respectively: https://ecocyc.org/gene?orgid=ECOLI&id=EG10025#tab=FTRS and https://ecocyc.org/gene?orgid=ECOLI&id=EG11306#tab=FTRS.

In summary, 914 proteins were updated by data showing at least one lysine that can be acetylated. Acetylation data were added to 2,065 distinct lysine residues in the proteome.

The preceding protein pages for AceF and LipA illustrate the ability of EcoCyc to capture the functions of substitution mutants in the Protein Features tab. For example, the page for AceF captures the fact that an H to C substitution at position 603 abolishes the catalytic activity of the protein (see the first feature table). A total of 6,792 such “mutagenesis variant” protein features are present in EcoCyc, although there must be additional such information in the experimental literature. EcoCyc contains 40,051 protein features in total (including the preceding 6,792), including, for example, enzyme active sites and metal ion binding sites.

#### Regulation of Gene Expression

Since 2017, a significant amount of new data related to specific promoters, regulatory interactions (RIs) and transcription units in *E. coli* K-12 has been published. This increase is reflected in new database objects and in modifications to existing objects as shown in Table 2. The largest number of modifications comes from enriching summaries and adding new evidence to existing objects.

**Table 2:** Summary of curation of the regulation of gene expression in EcoCyc between releases 21.1 and 24.5.

Object type in EcoCyc	New objects	Modified objects
Regulatory interaction (TF binding site)	1,331	640
DNA binding sites	1,161	568
Transcription units	188	679
Promoters	197	228
Proteins	13	402
Reactions	8	0
Terminators	64	74
Allosteric regulation of RNAP (ppGpp and DksA)	140	140

We have continued expanding the description of transcriptional regulation by including the binding of regulatory molecules directly to RNA polymerase. Examples are the allosteric regulation of RNA polymerase by ppGpp and DksA.

##### Regulatory Interactions Extracted From High-Throughput Experiments

As a result of the increasing *E. coli* K-12 literature involving the use of high-throughput technologies (HTs; Santos-Zavaleta et al., 2018), we have increased the number of DNA binding sites and their associated RIs (Table 2). Of the total number of new RIs, over 1,000 come from HT experiments with seven transcription factors. These RIs were identified by the authors through the combination of genome binding and expression profiling experiments, such as variants of chromatin immunoprecipitation (ChIP) and RNA-seq and microarray analyses, respectively (Table 3).

**Table 3:** Transcription factors and their regulatory interactions (RIs) extracted from high-throughput experiments.

Transcription factors	Number of curated RIs	Experimental strategy	References
ArcA	141	ChIP-chip and microarrays	Federowicz et al., 2014
ArgR	44	ChIP-exo	Cho et al., 2015
		Microarrays	Caldara et al., 2006
OmpR	12	gSELEX and microarrays	Shimada et al., 2015
CsiR	112	ChIP-seq and RNA-seq	Aquino et al., 2017
FNR	47	ChIP-chip and microarrays	Federowicz et al., 2014
Lrp	63	ChIP-chip and microarrays	Cho et al., 2008
	316	ChiP-seq and RNA-seq	Kroner et al., 2019
Nac	516	ChIP-seq and RNA-seq	Aquino et al., 2017

##### Redefinition of Basic Concepts in Gene Regulation

The conceptual data model used in EcoCyc to organize the knowledge about transcriptional regulation derives from the initial model by Jacob and Monod of the operon concept (Jacob and Monod, 1961). After 60 years of research with many technological advances before and after the explosion of HT methodologies in genomics, it was the time to revise the classic definitions to update them with our current knowledge on the regulation of transcription initiation in bacteria. Based on the consensus view of a group of experts (Mejía-Almonte et al., 2020), we have modified some aspects of modeling this knowledge in EcoCyc. For instance, a single promoter object was previously used to represent transcription start sites (TSSs) for RNA polymerase holoenzymes containing different sigma factors. Now, each of those TSSs belongs to a different promoter because each may be subject to different regulation even if the TSS is at exactly the same genome location (Mejía-Almonte et al., 2020). Conversely, given the known flexibility of RNA polymerase, one promoter may have more than one TSS within a region of five base pairs (Liu and Turnbough, 1994; Walker and Osuna, 2002; Winkelman et al., 2016). This limit is now being used in EcoCyc to add newly identified TSSs to known promoters. In particular, this is the case with experiments identifying TSSs and their associated transcription units from HT experiments (Yan et al., 2018; Ju et al., 2019).

### The *Escherichia coli* K-12 MG1655 GenBank File, U00096.3

EcoCyc has worked together with the original submitter, Dr. Guy Plunkett III, and staff from UniProt and NCBI to update the *E. coli* GenBank entry U00096.3, with the last update deposited on September 23, 2020. All genome annotation data within this entry, such as gene symbols, gene positions, and updated function names, are drawn directly from EcoCyc. Gene names are updated from the originally assigned “y-names” if a new name was assigned in the experimental literature. We encourage renaming “y-genes” with Demerec-style gene names (Demerec et al., 1966) once a function has been discovered. A brief summary on the history of the sequenced genome and guidelines for new gene names can be accessed on the following website: https://www.genome.wisc.edu/sequencing/k12.htm.

### New Tools in EcoCyc

#### Metabolic Network Explorer

**Figure 3:**
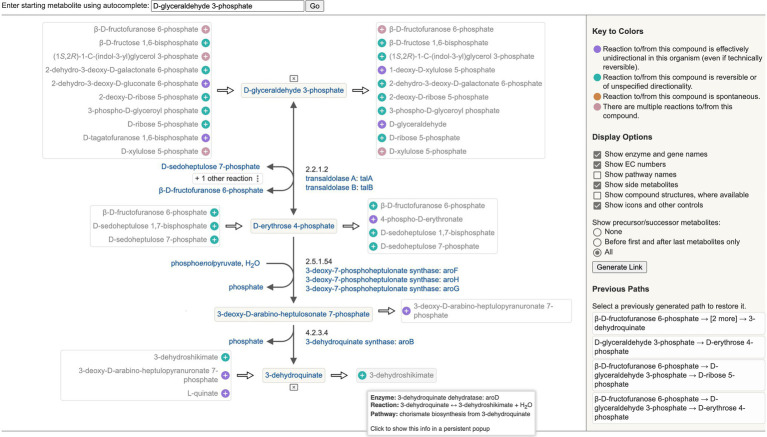
Example display of the Metabolic Network Explorer to explore *Escherichia coli* metabolism starting from the metabolite D-glyceraldehyde 3-phosphate.

The Metabolic Network Explorer (see website command Tools → Metabolism → Metabolic Network Explorer) is a new tool for interactively exploring the *E. coli* metabolic network around a metabolite of interest, as shown in Figure 3. The user specifies a starting metabolite, and the software displays that metabolite along with a full list of potential precursor and successor metabolites derived from the complete reaction network in EcoCyc. The tooltip for each potential precursor or successor metabolite lists all the reactions and enzymes that carry out the transformation and any pathways they belong to. After the user selects a precursor or successor metabolite to add it and its connecting reaction to a central path, that metabolite’s potential precursor and successor metabolites are added to the display. The user can continue to expand the central path in either or both directions by selecting metabolites at the start or end or the user can change the central path by selecting metabolites connected to internal metabolites. A list of paths previously generated in the current session is maintained to allow the user to quickly switch among them. The display includes several customization options such as whether to show metabolite structures or pathway names.

#### Circular Genome Viewer

**Figure 4:**
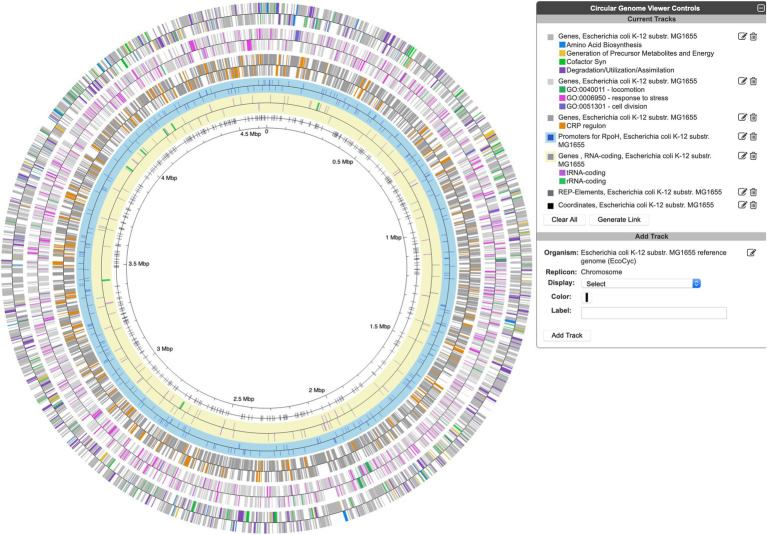
An example Circular Genome Viewer display, with tracks that showcase a variety of feature types, filtering and highlighting options, listed in order from the outermost circle inwards.

A new circular genome viewer (Tools → Genome → Circular Genome Viewer) provides a global view of the organization of the chromosome as a set of concentric circles (tracks) containing features (genes, promoters, binding-sites, and other extragenic sites) of interest. A given track can be filtered at the outset to only show features that match certain criteria (the available selection criteria depend on the feature type) or it can include a larger set of features; various selection criteria can be applied after the fact to highlight subsets of features. Possible feature types that can be displayed include genes, pseudogenes, promoters, transcription factor binding sites, REP elements, and others. The set of filtering and highlighting criteria for genes include product type (e.g., RNAs, enzymes, and transporters), name substrings, pathway classes, regulons, GO terms, and gene identifiers from an uploaded file. Figure 4 shows an example display with a variety of feature types and highlights. The circular genome viewer can also combine tracks from multiple strains or related species and highlight the orthologs between them.

### Revised Tools in EcoCyc

EcoCyc contains extensive web search options including a new command for searching for pseudogenes and different types of RNAs (website command Tools → Search → Genes, Proteins, or Tools → Search → RNAs → Search/Filter by type/subunits). We have also added a web-based search tool for searching for DNA and RNA sites of various types such as attenuators, riboswitches, phage attachment sites, and transposons (website command Tools → Search → Search DNA or mRNA sites).

We have upgraded the multiple-sequence alignment tools available for EcoCyc to use Clustal Omega (Sievers and Higgins, 2021) to compute alignments and MSA Viewer (Yachdav et al., 2016) to display the alignments (website command Analysis → Multiple Sequence Alignment).

**Figure 5:**
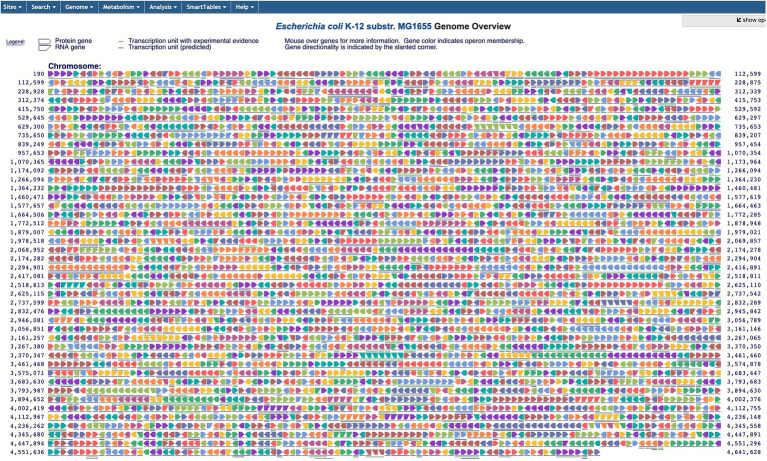
The Genome Overview diagram captures an entire genome with each arrow representing a single gene; neighboring genes that are part of the same operon are displayed in the same color. Arrow sizes are not to scale.

The Genome Overview diagram depicts the entire *E. coli* gene in a single screen (Figure 5 and website command Tools → Genome → Genome Overview). Each gene is shown as a single arrow with an arrowhead style distinguishing protein-coding genes from RNA-coding genes, and arrow direction indicating transcription direction. Adjacent genes drawn in the same color are within the same operon. We recently added the ability to search the diagram for genes by name or by substring (e.g., find all the genes whose name contains “arg”) and to highlight the search results on the diagram.

**Figure 6:**
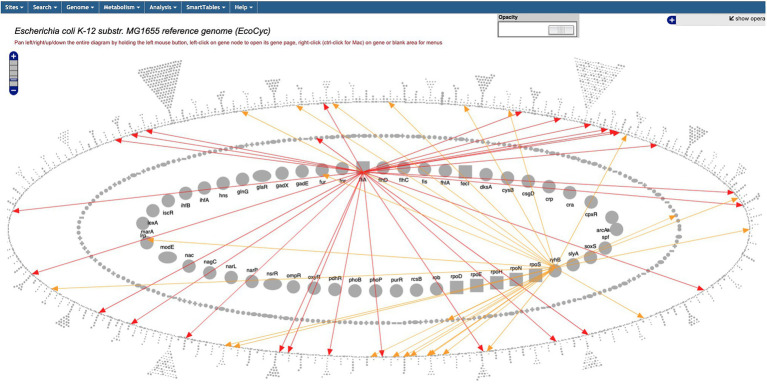
The Regulatory Overview diagram captures user-specified portions of a regulatory network. The user has specified the display of regulation by FliA (red) and RhyB (yellow).

The Regulatory Overview diagram depicts the *E. coli* regulatory network, more specifically, transcriptional regulation (including transcription factors and sigma factors), and translational regulation (including small RNAs). The diagram (Figure 6 and website command Tools → Genome → Regulatory Overview) is organized into three concentric ellipses; the inner ellipse depicts global regulatory genes, the middle ellipse depicts other regulatory genes, and the outer ring depicts genes that are not regulators. The diagram supports a variety of operations, including searching for genes by names and highlighting the regulators or regulatory targets of a given gene. A new command enables the user to output either the entire regulatory network or a subnetwork starting at a given gene to an ASCII file whose indentation describes the hierarchy of regulatory relationships.

The Cellular Overview diagram depicts the full *E. coli* metabolic and transport network (see website command Tools → Metabolism → Cellular Overview). All EcoCyc pathways are included, grouped by class, along with a section for reactions that have not been assigned to pathways. Transporters and other membrane proteins are shown on a schematic of the double membrane, with periplasmic reactions and proteins between the membranes. The diagram supports highlighting operations for genes, proteins, metabolites, reactions, and pathways using a variety of criteria. This diagram is also used by the Omics Viewer, in which omics data, such as transcriptomics or metabolomics data, are overlaid on the cellular overview to illustrate experimental results in a metabolic context. The Omics Viewer has also been substantially revamped to give the user extensive interactive control over the mapping of omics data values to colors, including the ability to selectively hide or show specified data ranges.

All three of the overview diagrams have been re-engineered to use modern, high-quality graphics that draw more rapidly and to provide real-time semantic zooming capabilities.

## Discussion

The EcoCyc database is unique in its extensive coverage of *E. coli* biology captured from a century of research. Ongoing manual curation enables the addition of new gene product functions and other important new research results, while the incorporation of new high-throughput datasets expands the types of data stored in the database. EcoCyc also welcomes user input. The “Provide Feedback” button on each data page can be used to submit information on new publications, to point out errors or omissions, and to suggest other improvements.

Future directions for EcoCyc include integrating EcoCyc with the *E. coli* whole cell model developed by the laboratory of Prof. M. Covert (Macklin et al., 2020) and improving the EcoCyc search and visualization tools.

## Data Availability Statement

Publicly available datasets were analyzed in this study. This data can be found at: www.ecocyc.org.

## Author Contributions

IK, SP, PK, AM, MK, JC-V, and AW: writing of manuscript. IK, AM, AS-Z, SG-C, VT, RC, and WO: EcoCyc curation. LM-R, CB-M, SP, MK, AK, and PM: EcoCyc data import. PS and RB: EcoCyc releases and website. SP, MK, WO, AK, PM, PS, and RB: Pathway Tools software development. PK, JC-V, and IP: guidance and oversight. PK and JC-V: funding. All authors contributed to the article and approved the submitted version.

## Conflict of Interest

The authors declare that the research was conducted in the absence of any commercial or financial relationships that could be construed as a potential conflict of interest.

## Publisher’s Note

All claims expressed in this article are solely those of the authors and do not necessarily represent those of their affiliated organizations, or those of the publisher, the editors and the reviewers. Any product that may be evaluated in this article, or claim that may be made by its manufacturer, is not guaranteed or endorsed by the publisher.

## Footnotes

^1^ https://ecocyc.org/ECOLI/NEW-IMAGE?type=PATHWAY&object=PWY-5838&detail-level=2
